# Right Aortic Arch and Kommerell's Diverticulum Repaired without Reconstruction of Aberrant Left Subclavian Artery

**DOI:** 10.1155/2013/840804

**Published:** 2013-04-24

**Authors:** Hiroshi Osawa, Daisuke Shinohara, Kouan Orii, Shigeru Hosaka, Shoji Fukuda, Okihiko Akashi, Hiroshi Furukawa

**Affiliations:** ^1^Division of Cardiovascular Surgery, Shimada General Hospital, Higashi-cho 5-3, Choshi, Chiba 288-0053, Japan; ^2^Department of Cardiovascular Surgery, National Center of Global Health and Medicine, Shinjuku, Tokyo 162-8655, Japan; ^3^Division of Cardiovascular Surgery, Ikegami General Hospital, Ota, Tokyo 146-8531, Japan; ^4^Department of Cardiovascular Surgery, Kawasaki Medical University, Kurashiki, Okayama 701-0192, Japan

## Abstract

Right aortic arch with Kommerell's diverticulum is a very rare situation. Surgical treatment is recommended for symptomatic patients or asymptomatic patients with a large diverticulum. However planning the strategy of operation is difficult without a 3D imaging. We report a case of a 57-year-old man with right aortic arch, Kommerell's diverticulum, and aberrant left subclavian artery. After a 3D-CT imaging, the patient underwent descending aortic replacement without reconstruction of aberrant left subclavian artery. After operation, there was no signs or symptoms of ischemia of the left arm. If the reconstruction of the aberrant subclavian artery was too difficult, closing its orifice is an acceptable decision. It has been found advantageous because of a decrease blood loss and a shorter cardiopulmonary bypass duration. If an ischemia of the arm is noticed, additional reconstruction will have to be considered. 3D-CT imaging was very useful to have a proper orientation and plan for the operative strategy.

## 1. Introduction

Right aortic arch with Kommerell's diverticulum (KD) is a very rare situation. Most patients with KD are asymptomatic; however the most serious issue in the course of aneurysm is its marked propensity towards rupture and dissection [1, 2]. As per earlier reports, all the patients who presented with rupture has died [1]. Therefore the surgical treatment of KD is indicated, before KD will rupture. However it is difficult to image and make a strategy of operation. So, we report the operating procedure and the evaluation method of KD using several views of 3D-CT imaging.

## 2. Case Presentation

**Figure 1:**
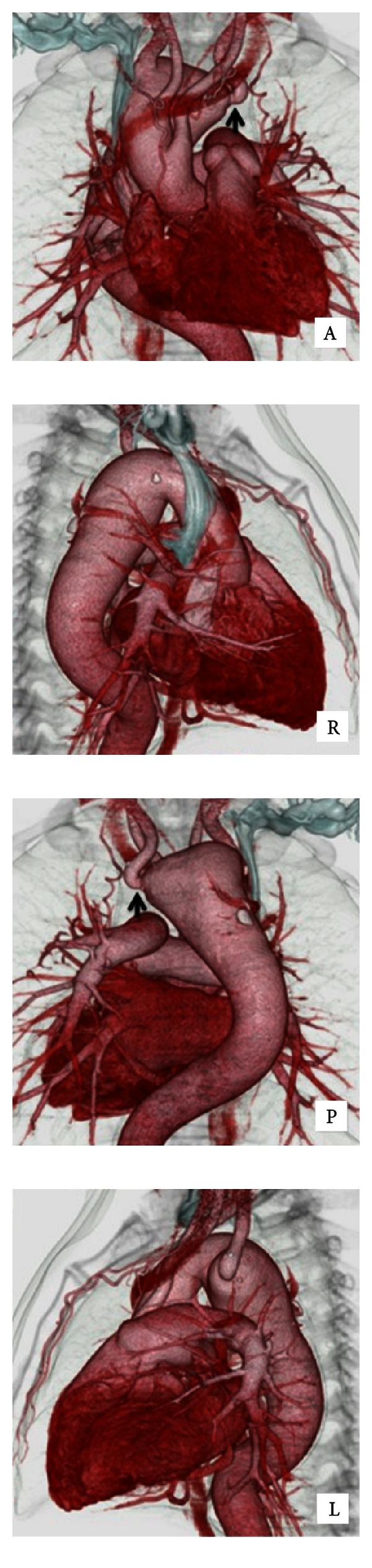
Enhanced CT showed right aortic arch, Kommerell diverticulum, and aberrant left subclavian artery. There was a 50% stenosis of the orifice of aberrant left subclavian artery (arrows).

A 57-year-old man had abnormal X-ray findings during routine medical checkup and an enhanced computed tomography (CT) showed right aortic arch, Kommerell diverticulum (KD), and aberrant left subclavian artery (ALSA). The maximum diameter of KD was 38 mm, and the aneurysm of aorta was 63 mm (Figure 1). CT revealed a 50% stenosis of the orifice of ALSA. Cerebral magnetic resonance imaging angiography showed hypoplasty of left vertebral artery. Operation was indicated because the diameter was critical, despite the fact that the patient had no symptoms due to KD. 

**Figure 2:**
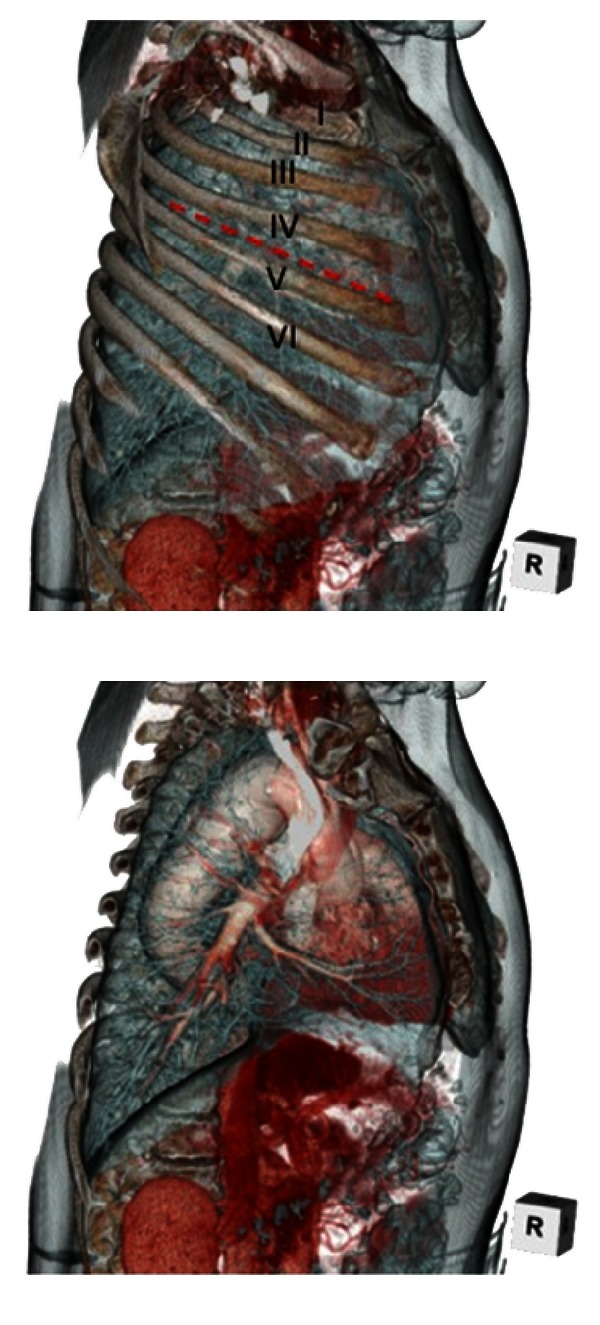
3D-CT imaging, scrolling from superficial to deep layer view with chest wall including the ribs, showed the surgical view and the optimal approach through a posterolateral thoracotomy with the fourth intercostal incision.

 After consideration of using 3D-CT imaging with the ribs (Figure 2), it was decided to go for a posterolateral thoracotomy approach through a fourth inters costal incision. 

 All the aneurysm repairs were performed using hypothermic cardiopulmonary bypass and an interval of hypothermic circulatory arrest. Cardiopulmonary bypass was established with descending aortic cannulation and bicaval drainage. The left heart was vented through the right upper pulmonary vein. The right phrenic, vagus, and recurrent laryngeal nerves were identified and protected. After establishing circulatory arrest, the aorta was incised and transected proximally just distal to the orifice of the right subclavian artery. Descending aorta with KD was replaced using one branched graft for the reconstruction of ALSA. However ALSA was not able to be reconstructed anatomically and ligated because ALSA was too deep and difficult to make anastomosis. Rewarming was initiated after completion of the proximal graft to aorta anastomosis. Distal graft to aorta anastomosis was performed under descending aortic clamping and blood supplying from proximal anastomosed graft and descending aorta. 

**Figure 3:**
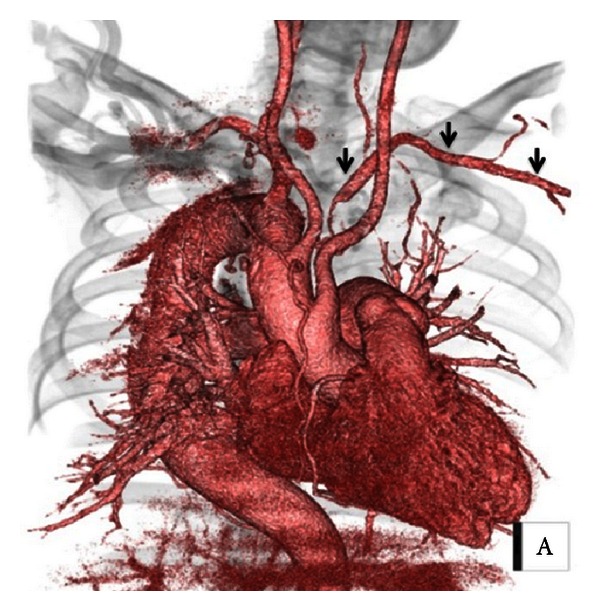
Postoperative CT revealed enhanced ALSA (arrows) clearly without delay, which was supplied with blood from many branches through very narrow and invisible collateral arteries.

 After operation, there were no symptoms of an ischemia of the left arm. The systolic blood pressure of the right arm and the left arm was 110 mmHg and 70 mmHg, respectively. Postoperative CT revealed enhanced ALSA clearly without delay, which was supplied with blood from many branches through the narrow and invisible collateral arteries (Figure 3). 

## 3. Discussion

Kommerell's diverticulum and aberrant subclavian artery can be discovered accidentally in asymptomatic children or adults, but sometimes they are associated with complications, such as compression of adjacent structures, dissection or rupture. Standard management of KD has not been established because of the rarity of this anomaly [2]. Generally speaking, surgical intervention is recommended in symptomatic patients or asymptomatic patients with a large diverticulum.

 Several reports recommend an operative strategy of descending aortic replacement and anatomical or extra anatomical reconstruction of aberrant subclavian artery [2, 3] for KD. Posterolateral thoracotomy provides excellent exposure of the aortic arch and descending aorta, which allows reconstruction of aneurysm including KD and aberrant subclavian artery repair. 

 With regard to the reconstruction of the aberrant subclavian artery, in situ repair is optimal. Esposito and colleagues have reported significant ischemic complications such as coldness, rest pain, and fingertip necrosis in 64% of patients treated without restoration of the blood flow to the arm [4]. 

 However, if the reconstruction of the aberrant subclavian artery was too difficult, to close an orifice is acceptable. Because, when the ischemia is noticed after aortic procedure, to make an extra anatomical reconstruction like a subclavian to subclavian artery bypass could be considered. It is one of the options of operation of an aneurysm with KD. It has some advantages, for example, decrease of blood loss and cardiopulmonary bypass time. We recommend this method especially for cases where the orifice of ALSA is noticed over the vertebra. Ota and colleagues reported that ligation of ALSA would be acceptable when a stenosis of orifice of ALSA was noticed, under observation of intraoperative blood pressure of both arm, and confirmation of unchanged blood pressure of left arm [3]. There is a possibility that the stenosis of ALSA might bring a preparation for collateral development. Kouchoukos also recommend a carotid to subclavian artery bypass in patients where direct continuity between the descending thoracic aorta and the distal subclavian artery could not be easily established [2].

 To make the right strategy of operation, preoperative 3D-CT identification of anomalous structures is very useful, especially in rare cases such as the one with KD and a right aortic arch [5]. 3D-CT, especially scrolling from superficial to deep layer view with the chest wall including ribs (Figure 2), was very useful for us to have a proper orientation and to make an operative strategy.

## 4. Conclusion

Kommerell's diverticulum could be repaired without reconstruction of the aberrant left subclavian artery. It was found advantageous because of the decrease of blood loss and the shorter cardiopulmonary bypass time. If an ischemia of arm is noticed, additional reconstruction will have to be considered.

## Supplementary Material

The movie of 3D-CT imaging that scrolling from superficial to deep layer view with chest wall including the ribs, showed the surgical view of right aortic arch and Kommerell's diverticulum.

## References

[1] Austin EH, Wolfe WG (1985). Aneurysm of aberrant subclavian artery with a review of the literature. *Journal of Vascular Surgery*.

[2] Kouchoukos NT, Masetti P (2007). Aberrant subclavian artery and Kommerell aneurysm: surgical treatment with a standard approach. *Journal of Thoracic and Cardiovascular Surgery*.

[3] Ota T, Okada K, Takanashi S, Yamamoto S, Okita Y (2006). Surgical treatment for Kommerell's diverticulum. *Journal of Thoracic and Cardiovascular Surgery*.

[4] Esposito RA, Khalil I, Galloway AC, Spencer FC (1988). Surgical treatment for aneurysm of aberrant subclavian artery based on a case report and a review of the literature. *Journal of Thoracic and Cardiovascular Surgery*.

[5] Nakada T, Sakao Y, Gorai A, Uehara H, Mun M, Okumura S (2012). Two patients of left lung cancer with right aortic arch: review of eight patients. *General Thoracic and Cardiovascular Surgery*.

